# Sequencing bias: comparison of different protocols of MicroRNA library construction

**DOI:** 10.1186/1472-6750-11-48

**Published:** 2011-05-11

**Authors:** Geng Tian, ShangQuang Gan, XuYang Yin, Hong Luo, XiaoHong Xu, Lars Bolund, XiuQing Zhang, Ning Li

**Affiliations:** 1 Beijing Institute of Genomics, Chinese Academy of Science, Beijing 101300, China; 2 Key Labs of Sheep Breeding and Reproduce, Xinjiang Academy of Agricultural and Reclamation Science, Shihezi 832000, China; 3 Genome Research Institute, ShenZhen University Medical School, ShenZhen 518000, China; 4 Beijing Genomics Institute, Shenzhen 518000, China; 5 Insitute of Human Genetics, University of Aarhus, Aarhus DK-8000, Denmark; 6 State Key Laboratories for Agrobiotechnology, College of Biological Science, China Agriculture University, Beijing 100193, China

## 

After publication of this work [1], we noted that we inadvertently failed to add our collaborators ShangQuang Gan and Ning Li to the complete list of authors.

## Authors' contributions

GT participated in the design of the study. SQG selected and prepared the samples. XYY participated in the design of the study, drafted the manuscript, and carried out the qRT-PCR studies. HL and XHX carried out miRNA libraries' construction for cloning and SBS sequencing. XQZ and NL participated in the design of the study. LB participated in the interpretation of data. All authors revised the manuscript and approved the final version to be published.
